# Development and Control of a Pneumatic-Actuator 3-DOF Translational Parallel Manipulator with Robot Vision †

**DOI:** 10.3390/s19061459

**Published:** 2019-03-25

**Authors:** Lian-Wang Lee, Hsin-Han Chiang, I-Hsum Li

**Affiliations:** 1 Department of Mechanical Engineering, National Chung Hsing University, No. 145, Xingda Road, South District, Taichung City 40227, Taiwan; leelw@dragon.nchu.edu.tw; 2 Department of Electrical Engineering, National Taiwan Normal University, No 162, Section 1, Heping East Road, Taipeu City 106, Taiwan; hsinhan@ntnu.edu.tw; 3 Department of Mechanical and Electro-Mechanical Engineering, Tamkang University, No. 151, Yingzhuan Road, Tamsui District, New Taipei City 25137, Taiwan

**Keywords:** interval type-2 fuzzy systems, path tracking control, parallel manipulator, rodless pneumatic actuator, robotic manipulators, robot vision

## Abstract

A vision-based three degree-of-freedom translational parallel manipulator (TPM) was developed. The developed TPM has the following characteristics. First, the TPM is driven by three rodless pneumatic actuators and is designed as a horizontal structure to enlarge its horizontal working space to cover a conveyor. Then, a robot-vision system (including a webcam mounted on the TPM) collects images of objects on the conveyor and transfers them through the LabVIEW application programming interface for image processing. Since it is very difficult to achieve precise position control of the TPM due to the nonlinear couplings among the robot axes, feedback linearization is utilized to design an adaptive interval type-2 fuzzy controller with self-tuning fuzzy sliding-mode compensation (AIT2FC-STFSMC) for each rodless pneumatic actuator to attenuate nonlinearities, function approximation errors, and external disturbances. Finally, experiments proved that the vision-based three degree-of-freedom TPM was capable of accurately tracking desired trajectories and precisely executing pick-and-place movement in real time.

## 1. Introduction

Robotic manipulators are efficient at picking, placing, and assembling objects and at tracking movements. With respect to their kinematic structures, robotic manipulators are generally divided into two types, namely a serial and a parallel type. A serial manipulator is designed as a series of links, which are sequentially connected by actuated joints, from a base to an end-effector. This arm-like structure is highly flexible in large-scale operations. However, the serially linked mechanism provides low positioning accuracy because the error for each joint and link is superimposed and the stiffness is insufficient for heavy loads. For a parallel manipulator (PM), several independent kinematic chains are connected by a moving platform, termed an end-effector, and a fixed base platform, while the actuators are located on or near the fixed base. PMs have many advantages, mainly because the load is shared by the several links connecting the moving platform to the base. Compared to the serial type of manipulators, the advantages of PMs also include higher stiffness, higher load-carrying capacity, and being able to achieve superior precision with higher speed and acceleration [1]. In addition, for PMs, the error in the position for each single manipulator is the average of errors for all manipulators and is not cumulative. The disadvantages of parallel-type robotic manipulators are that they allow only a limited workspace, require complex kinematic analysis, and are extremely difficult to control [2]. However, the increasing demand for high-speed operation, high precision, and increased stiffness means that PMs are increasingly important to industrial automation.

The first PM was designed as an amusement device by James E. Gwinnett in 1928. Further, a six degree-of-freedom (6-DOF) platform was developed by Stewart [3] and was called the Stewart platform. It has numerous applications in flight simulators and medical applications [1,4]. However, a 6-DOF PM has problems in complex kinematic analysis and motion coupling between axes and, thus, is not ideal for many applications. A PM with a relatively simple structure, such as a three degree-of-freedom (3-DOF) PM, has the inherent advantages of parallel mechanisms but offers additional cost reductions in manufacturing and operations. Hence, development of simple-structure PMs has been the focus of many researchers. In 1988, the DELTA robot with 3-DOF was designed by the research team of Clavel [5], and it was demonstrated to be suitable for industrial applications [6]. Since then, researchers have been involved in developing 3-DOF PMs with different structures and configurations, such as spherical 3-DOF mechanisms, 3-RPS PMs, and orthoglide parallel robots [6,7]. In addition, parallel manipulators have advantages of carrying a high payload with high-performance output motion. In 2017, Zi et al. [8] developed a winding hybrid-driven 3-cable PM, in which the three cables are applied to suspend an end-effector. By controlling the cables and utilizing gravitational force, the end-effector is able to move within a large workspace. Zi et al. used an adaptive fuzzy sliding mode controller for dynamic control compensation and verified the trajectory tracking through experimental studies. A kinematic calibration method was proposed by Qian et al. in [9] for cable-driven PM to improve the efficiency of calibration measurement. Qian et al. verified its accuracy and efficiency according to cable length through numerical simulation. However, the manufacturing of 3-DOF PMs encounters difficulties in the trajectory tracking control due to complicated dynamic and kinematic modeling [10]. To the present, joint space control and task space control are the most used strategies in controlling PMs. In the task space control, the forward kinematic problem can be effectively solved by using the measured lengths of the PM links. As compared to the joint speed control, the more precise position of the PM end-effector can be performed by using vision and position sensors [11,12]. In 2011, Chiang et al. [13] developed a 3-prismatic-universal-universal (3-PUU) PM that is driven by three vertical-axial pneumatic actuators (PAs) and used servo control for path tracking. For robotic arms, the robot vision provides extensive information for controlling the positioning of the end-effector for object handling, thereby, improving the system’s accuracy, flexibility, and self-sufficiency [14]. Therefore, vision-based robotic arms have recently attracted considerable attention for industrial applications. Papanikolopoulus et al. [15] and Oh et al. [16] et al. presented visual servoing control, which uses visual information to control the position of the robot’s end-effector relative to a target object. Peurla et al. [17] presented a low-cost vision-guided robot arm to place grids on films; that robot arm can reach a 90% success rate in pick-and-place experiments. In [18], joint space control for a three-axial pneumatic PM was developed with a stereo vision 3D position measurement system; the desired trajectory in task space was obtained by solving the inverse kinematic problem. In [19], vision-based control was investigated for the path tracking control of a 3-DOF translational PM (TPM) in which the pose of the end-effector was measured by a stereo vision system. 

Recently, three types of actuators have been widely utilized for driving PMs, namely, electric, hydraulic, and pneumatic. Among them, PAs have many advantages over other types of driving actuators, such as a cost-effective actuation, the cleanliness of the working environment, ease of maintenance, and rapid movement and reactions. However, the complexity of the trajectory tracking design in PMs easily raises while using PAs, which mainly originate from the compressibility of air, different regimes of air flow through valves, and the dynamic behavior of the friction forces of pneumatic cylinders. Inthis regard, various control strategies have been developed by researchers for the trajectory tracking control of 3-DOF TPMs. In [18], the performance levels of different control algorithms based on proportional-integral-derivative (PID) and adaptive control design were experimentally evaluated on a 3-DOF planar PM. In addition, an adaptive dynamic control scheme was proposed in [19] and implemented for a 3-DOF PM to conduct the experimental study. A convex synchronized control method was used in the trajectory tracking control of a 3-DOF planar PM and evaluated under several experimental conditions [20]. In [21], a dynamic control scheme of parallel kinematic mechanism based on a model-based approach was proposed and experimentally validated on a 3-DOF PM. So far, the rodless PA (RPA) is an important type of PM, which has been widely used in robotics and industrial automation. Rodless pneumatic actuators (RPAs) have numerous advantages, such as low cost, low maintenance requirements, safe operation, ease of operation, simple structure, and high power-to-weight ratio [22,23]. It is well known that the servo position control technique plays a key role in the application of pneumatic servo systems [24]. The smooth and accurate positioning of RPAs for complex control tasks remains challenging due to the nonlinearities of RPAs, which majorly come from low stiffness and compressibility of air, large friction forces, dead zones, and varying rates of air flow through servo valves [25]. These nonlinear characteristics of RPAs also cause a large number of difficulties for modeling and control. To deal with this problem, researchers [26,27,28,29] have recently developed advanced models and nonlinear control strategies to improve the performance of RPAs. Tsai [30] presented a sliding-mode controller (SMC) for an RPA and demonstrated that the RPA had favorable robustness and a high tolerance for uncertainties [30]. Lee et al. [31] also proposed an SMC that used functional approximation with H∞ tracking performance for RPA trajectory tracking. Zhao et al. [32,33] utilized an SMC scheme with an active disturbance rejection controller to deal with strong static friction and unknown disturbances in pneumatic servo position systems. However, the detailed dynamic model for RPAs is too difficult to be identified due to complex nonlinearities and time-varying characteristics of RPAs. Motivated by the previous discussions, this paper presents an intelligent model-free control system for RPAs. To improve RPA control performance, the proposed control system employs an adaptive interval type-2 fuzzy approximation technique to design an equivalent controller for a conventional SMC and combines a self-tuning scheme using the fuzzy SMC approach to compensate for function approximation errors, unmodeled dynamics, and disturbances. The proposed control scheme does not require any system dynamic model for controller design, is free from chattering, has been proven to be stable for tracking control, and is robust against uncertainties.

The three purposes of this paper are as follows. The first purpose is to develop, from scratch, a complete mechatronic design of a low-cost 3-DOF PM to effectively control translational motion. The second purpose is to develop a test-rig of a vision-based PM-driven TPM, which allows the implementation and testing of dynamic control schemes for the 3-DOF TPM. The third purpose is to develop robot vision and implement pick-and-place operations for the 3-DOF PM. For these purposes, a 3-DOF TPM, driven by three horizontal-axial RPAs with the associated proportional directional control valve (PDCV), was developed for three-dimensional (3D) path tracking control. The assembly configuration of the RPAs arranges the PM as a horizontal structure, which offers a large horizontal working space. In terms of the design of the controller, the RPA model in the three horizontal motion axes is first derived. A typical RPA suffers from bounded unknown parameters and external disturbances; to allow 3D path tracking control for the moving platform, this study used feedback linearization techniques, an interval type-2 fuzzy system, sliding-mode control theory, and an adaptive control scheme to control the positions for the three horizontal RPAs. To locate an object, images of the object are collected by a webcam and are transferred to LabVIEW through an application programming interface (API) with image processing toolkits to locate the object’s coordinates for the system. 

In this paper, there are main superiority and innovations in the developed vision-based 3-DOF TPM, which are:

- To develop a low-cost and high-reliability TPM, we use a rodless pneumatic actuator (RPA), which has many superiorities in its low-cost, low maintenance requirements, safe operation and simple structure, to drive the TPM.
- The presented adaptive interval type-2 fuzzy controller with self-tuning fuzzy sliding-mode compensation (AIT2FC-STFSMC) effectively attenuates the nonlinearities in the TPM, which come from two parts: (1) the pneumatic cylinder leads to its low stiffness and compressibility of air and large friction forces; and (2) the use of the valve leads to dead zones and varying rates of air flow through servo valves.
- The assembly configuration of the RPAs arranges the PM as a horizontal structure, which offers a large horizontal working space, and a soft pneumatic gripper installed on the end-effector of the TPM is able to easily grip an object.
- The develop vision-based 3-DOF TPM has the ability to accurately execute pick-and-place movement in real time.

The remainder of this paper is organized as follows. In Section 2, the mechanical parts of the RPA-driven 3-DOF TPM and its experimental setup are described. Further, mathematical models for representations of the used RPA with cylinders and pressure dynamics are developed in Section 3. The control system design with the stability analysis is given in Section 4. Section 5 introduces the robot-vision technique with the LabVIEW API, and three experiments are presented in Section 6 to investigate the trajectory tracking performance and pick-and-place operation. Finally, Section 7 concludes this paper.

## 2. Test Rig Layout of 3-DOF TPM

**Figure 1:**
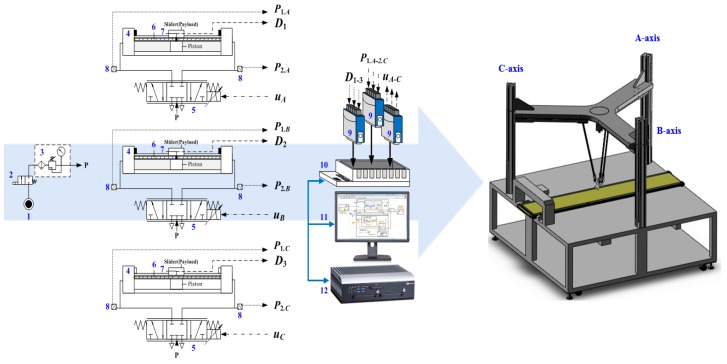
Test rig layout of the rodless pneumatic actuator (RPA)-driven three degree-of-freedom (3-DOF) translational parallel manipulator (TPM). 1. Pressure source; 2. Solenoid valve; 3. Air preparation unit; 4. Rodless pneumatic actuator; 5. Proportional directional control valve; 6. Linear encoder; 7. Scanning head; 8. Pressure sensor; 9. Interface card; 10. Compact-RIO embedded system; 11. LabVIEW software; 12. Industrial PC.

**Figure 2:**
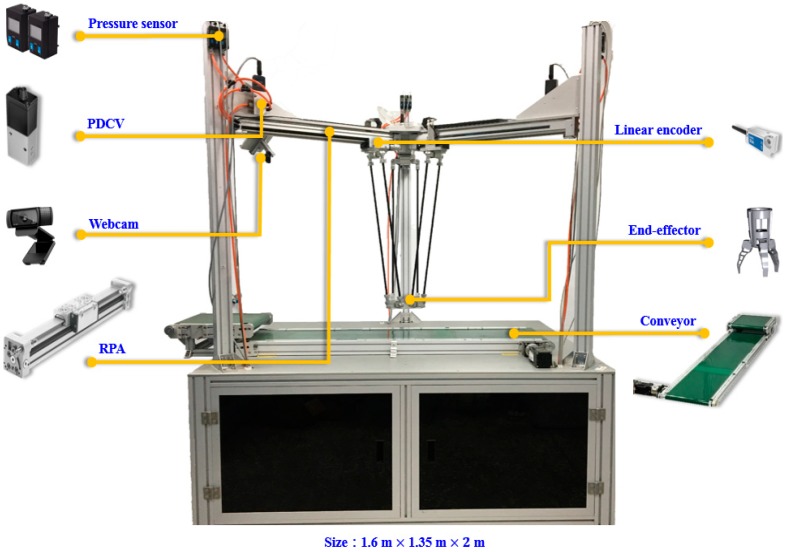
Photo of the RPA-driven 3-DOF TPM.

In this section, the geometrical configuration of the constructed RPA-driven 3-DOF TPM is first introduced. The layout of the 3-DOF TPM is shown in Figure 1, and its photo is shown in Figure 2. A PC-based control unit labeled 12 in Figure 1, which is installed inside the control box under the fixed platform, controls the system. This RPA-driven 3-DOF TPM communicates through analog-to-digital and digital-to-analog converter (AD/DA) interface cards. Three RPAs with 25-mm-diameter pistons are horizontally installed under the parallel links at 120 degrees to each other to move the platform in three dimensions. Three Festo-mode DGC-25-500 RPAs produce horizontal motion in this design; each RPA has a 500-mm stroke. For each horizontal axis, a Festo 5/3 MPYE-5-1/8-HF-010-B PDCV regulates the flow of air into the cylinder. This device is crucial to controlling the PM. The voltage supplied to each PDCV is at 24 V, and the control voltage is in the range of 0 to 10 V. The maximum recommended nominal flow is at 700 L/min. In this system, the pressure source is set at 6 bar. The two pressure levels in the two cylinder chambers are measured by two Festo model SDE1 pressure sensors, which are installed on the two ports of the cylinder. A PC-based controller provides a control signal that regulates the PDCVs to drive the horizontal RPAs through a D/A interface that operates at a sampling frequency of 200 Hz. To accurately measure the position of each horizontal RPA, three linear encoders with a resolution of 1 μm are installed on the RPAs. The position signals are sent to the PC-based controller through a counter card. For robot vision, a Logitech C920 webcam mounted on the support frame is used to capture images of each object.

## 3. Analysis of Kinematics

Typically, there are two parts for manipulator kinematics analysis, namely inverse kinematics and forward kinematics. From the analysis of inverse kinematics, a set of actuated joint variables can be identified to achieve a targeted position and orientation for the end-effector of TPM. In the forward kinematics, the position and orientation of the end-effector can be determined from the given actuated joint coordinates of TPM. This study employs the vector-loop closure equation [33,34] to derive both of the inverse and forward kinematics in this section. In the beginning, the desired 3D path profiles can be converted to the trajectory profiles for each of the three RPAs by using inverse kinematics. With the connection of three RPAs in a parallel mechanism with a link and RPA-driven joints, the end-effector mounted on a movable platform can, thus, be driven to perform 3-D motions while controlling each RPA to track the associated trajectory profile. 

### 3.1. Inverse Kinematic Analysis

**Figure 3:**
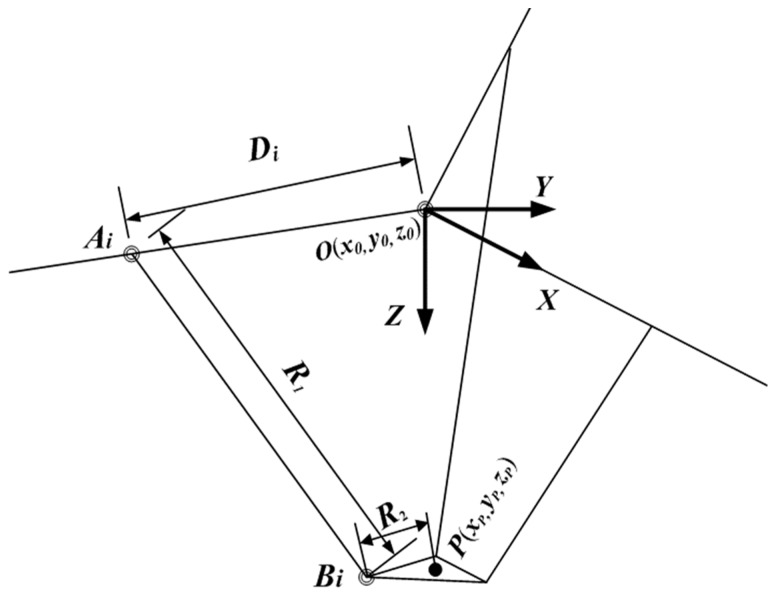
Schematic of the 3-DOF TPM.

Initially, two coordinate frames are investigated for the RPA-driven 3-DOF TPM to analyze the kinematic model, as shown in Figure 3. The first frame is a static Cartesian coordinate frame, $O(x_{0,}y_{0},z_{0})$, which is fixed at the center of the base, whereas a mobile Cartesian coordinate frame, $P(x_{p,}y_{p},z_{p})$, is assigned to the center of the mobile platform. In Figure 3, $A_{i}$ indicates the joints located at the center of the base and $B_{i}$ represents the passive joints of the moveable platform, (i=1,2,3). Since three links are installed between the mobile platform and the fixed platform, the lengths of the link $R_{1}$ and $R_{2}$ in Figure 3 can be expressed as:

(1) $$R_{1}=\bar{A_{1}B_{1}}\;=\bar{A_{2}B_{2}}=\bar{A_{3}B_{3}},\;R_{2}=\bar{B_{1}P}=\bar{B_{2}P}=\bar{B_{3}P}$$

**Figure 4:**
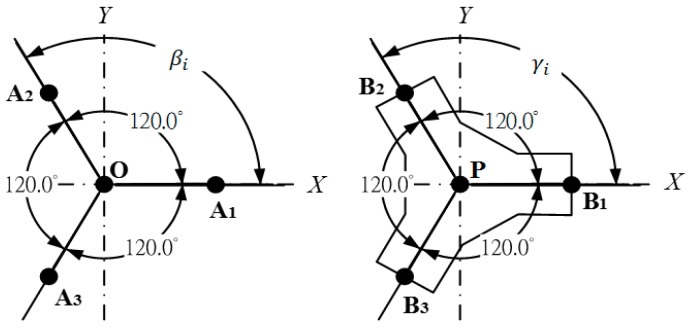
Schematic of mobile and fixed platforms for the 3-DOF TPM.

Let $C_{A_{i}}$ be the position of the point $A_{i}$, and let $C_{B_{i}}$ be the position of the point $B_{i}$; then, it follows that

(2) $$C_{Ai}=\left\{\begin{array}{l} D_{i}\times \cos\beta_{i}\\ D_{i}\times \sin\beta_{i}\\ 0 \end{array}\right\}$$

and

(3) $$C_{B_{i}}=C_{P}+C_{PB_{i}}=\left\{\begin{array}{l} x_{p}+R_{2}\times \cos\gamma_{i}\\ y_{p}+R_{2}\times \sin\gamma_{i}\\ z_{p} \end{array}\right\}$$

where $D_{i}$ represents the linear displacement of *i*th RPA that is also the cylinder piston position of *i*th limb; $\gamma_{i}$ and $\beta_{i}$ are computed as $\beta_{i}=\gamma_{i}=(i-1)\times 120^{{}^{\circ}}$, as displayed in Figure 4. According to Equations (1)–(3), one can calculate

(4) $${\bar{C_{Ai}C_{Bi}}}^{2}=R_{1}^{2}$$

Equation (4) can be further expressed as:

(5) $$\begin{array}{l} D_{i}^{2}-D_{i}\times 2[\left(x_{p}+R_{2}\times \cos\gamma_{i}\right)\times \cos\beta_{i}+\left(y_{p}+R_{2}\times \sin\gamma_{i}\right)\times \sin\beta_{i}]\\ +{\left(x_{p}+R_{2}\times \cos\gamma_{i}\right)}^{2}+{\left(y_{p}+R_{2}\times \sin\gamma_{i}\right)}^{2}+z_{p}^{2}-R_{1}^{2}=0. \end{array}$$

Let $U_{i}$ and $V_{i}$ be defined as:

(6) $$U_{i}=\left(x_{p}+R_{2}\times \cos\gamma_{i}\right)\times \cos\beta_{i}+\left(y_{p}+R_{2}\times \sin\gamma_{i}\right)\times \sin\beta_{i}$$

and

(7) $$V_{i}={\left(x_{p}+R_{2}\times \cos\gamma_{i}\right)}^{2}+{\left(y_{p}+R_{2}\times \sin\gamma_{i}\right)}^{2}+z_{p}^{2}-R_{1}^{2}=0$$

Then, substituting Equations (6) and (7) into Equation (5), the following kinematics of each RPA can be yielded.

(8) $$D_{i}=U_{i}\pm \sqrt{U_{i}^{2}-V_{i}}$$

Notably, Equation (8) has two solutions for each actuator. According to the current assembly of the mechanism, only the positive square root solution is the feasible condition while the actuator is translational from outside to inside. Consequently, the inverse kinematics equation for each RPA of the 3-DOF TPM can be found as:

(9) $$D_{i}=U_{i}+\sqrt{U_{i}^{2}-V_{i}}$$

### 3.2. Forward Kinematic Analysis

The objective of forward kinematics is to acquire the 3-D pose information for the end-effector. If given $D_{i}$, then $C_{A_{i}}$ and $C_{B_{i}}$ can be calculated by

(10) $$C_{Ai}=\left\{\begin{array}{l} (D_{i}-R_{2})\times \cos\beta_{i}\\ (D_{i}-R_{2})\times \sin\beta_{i}\\ 0 \end{array}\right\}$$

and

(11) $$C_{Bi}=\left\{\begin{array}{l} x_{p}+(R_{2}-R_{2})\times \cos\gamma_{i}\\ y_{p}+(R_{2}-R_{2})\times \sin\gamma_{i}\\ z_{p} \end{array}\right\}=\left\{\begin{array}{c} x_{p}\\ y_{p}\\ z_{p} \end{array}\right\}$$

Substituting Equations (10) and (11) into Equation (4) yields

(12) $${\left\{\begin{array}{l} x_{p}-(D_{i}-R_{2})\times \cos\beta_{i}\\ y_{p}-(D_{i}-R_{2})\times \sin\beta_{i}\\ z_{p} \end{array}\right\}}^{2}-R_{1}^{2}=0$$

Expressing the vectors in Equation (12) as:

(13) $$x_{p}^{2}+{\left(D_{i}-R_{2}\right)}^{2}\times \cos^{2}\beta_{i}-2x_{p}\left(D_{i}-R_{2}\right)\cos\beta_{i}+{y_{p}}^{2}+{\left(D_{i}-R_{2}\right)}^{2}\sin^{2}\beta_{i}-2y_{p}\left(D_{i}-R_{2}\right)\sin\beta_{i}+z_{p}^{2}-R_{1}^{2}=0$$

Let $(D_{i}-R_{i})=Q_{i}$, *i* = 1, 2, and 3, Equation (13) can then be rewritten as:

(14) $$x_{p}^{2}+Q_{1}^{2}-2x_{p}Q_{1}+y_{P}^{2}+z_{P}^{2}-R_{1}^{2}=0$$

(15) $$x_{p}^{2}+\frac{1}{4}Q_{2}^{2}+x_{p}Q_{2}+y_{P}^{2}+\frac{3}{4}{Q_{2}}^{2}-\sqrt{3}y_{p}Q_{2}+z_{P}^{2}-R_{1}^{2}=0$$

and

(16) $$x_{p}^{2}+\frac{1}{4}Q_{3}^{2}+x_{p}Q_{3}+y_{P}^{2}+\frac{3}{4}{Q_{3}}^{2}+\sqrt{3}y_{p}Q_{3}+z_{P}^{2}-R_{1}^{2}=0$$

Therefore, from Equations (14)–(16) the forward kinematics is described as follows:

(17) $$x_{p}=\frac{y_{p}\sqrt{3}Q_{2}+Q_{1}^{2}-Q_{2}^{2}}{2Q_{1}+Q_{2}}$$

(18) $$y_{p}=\frac{-\left[\left(Q_{3}^{2}-Q_{1}^{2}\right)\left(2Q_{1}+Q_{2}\right)+\left(Q_{1}^{2}-Q_{2}^{2}\right)\left(2Q_{1}+Q_{3}\right)\right]}{2\sqrt{3}(Q_{1}Q_{2}+Q_{2}Q_{3}+Q_{1}Q_{3})}$$

and

(19) $$z_{p}=-\sqrt{R_{1}^{2}-{\left(x_{p}-Q_{1}\right)}^{2}-y_{p}^{2}}$$

## 4. Analysis of the Design and Stability of the Controller

### 4.1. Dynamic Model of the RPA

In the RPA, the orifice area of the PDCVs can be controlled by the input air flow. With the pressure difference resulted from two chambers of the cylinder, the RPA can be moved in the desired position. According to the analysis in [35], the dynamic model of RPA can be derived while considering the dynamics and the mass flow rate of PDCV, the continuity equation, and the motion equation. Thus, the nonlinear model of the RPA can be presented in the form of a fourth-order nonlinear system as [35]:

(20) $$\begin{array}{l} {\dot{x}}_{1}=x_{2}\\ {\dot{x}}_{2}=\frac{1}{M}\left[-K_{f}x_{2}-K_{s-c}(x_{1})S(x_{2},x_{3},x_{4})+A(x_{3}-x_{4})\right]\\ {\dot{x}}_{3}=\frac{-k\left[x_{2}x_{3}-\frac{RT_{S}}{A}C_{d}C_{0}w\hat{f}(x_{3},P_{s},P_{e})u\right]}{x_{1}+\Delta }\\ {\dot{x}}_{4}=\frac{k\left[x_{2}x_{4}-\frac{RT_{S}}{A}C_{d}C_{0}w\hat{f}(x_{4},P_{s},P_{e})u\right]}{l-x_{1}+\Delta } \end{array}$$

where $x_{1}=x$, which is the piston position, $x_{2}=\dot{x}$; $x_{3}=P_{a}$, which is the pressure in chamber *a*; $x_{4}=P_{b}$, which is the pressure in chamber b; the control input u indicates the spool displacement of PDCV; $K_{s-c}(x_{1})$ denotes a combination of the static and dynamic frictions; w denotes a port width of the PDCV; l denotes a stroke of the RPA; Δ is the general residual chamber volume; R is the universal gas constant; $T_{s}=293\;K$ is the supply temperature; $C_{0}$ is the flow constant, and $C_{d}=0.8$ is the discharge coefficient. For the convenience of the analysis, the following function is introduced:

(21) f˜(pr)={1,PatmPu<pr≤CrCk[pr2k−pr(k+1)k]12,Cr<pr<1

where $P_{atm}$ is the atmospheric pressure; $p_{r}=P_{d}/P_{u}$ is the ratio between the downstream and the upstream pressures at the orifice; k=1.4 is the specific heat constant; $C_{r}={\left(2/k+1\right)}^{k/(k+1)}=0.528$, $C_{k}=\sqrt{2/(k-1)\times {\left(k+1/2\right)}^{\left(k+1)/(k-1\right)}}=3.864$. It can be shown that the function $\tilde{f}(\cdot )$ and its derivative are continuous with respect to $p_{r}$. According to Equation (21) the functions $\hat{f}(x_{3},P_{s},P_{e})$ and $\hat{f}(x_{4},P_{s},P_{e})$ in Equation (20) are defined as:

(22) $$\hat{f}(x_{3},P_{s},P_{e})=\left\{ \begin{array}{l} P_{s}\tilde{f}\left(\frac{x_{3}}{P_{s}}\right)/\sqrt{T_{s}},\;chamber=a\\ x_{3}\tilde{f}\left(\frac{P_{e}}{x_{3}}\right)/\sqrt{T_{a}},\;chamber=b \end{array}\right.$$

and

(23) $$\hat{f}(x_{4},P_{s},P_{e})=\{\begin{array}{c} x_{4}\tilde{f}\left(\frac{P_{e}}{x_{4}}\right)/\sqrt{T_{b}},chamber\begin{array}{c} = \end{array}a,\\ P_{s}\tilde{f}\left(\frac{x_{4}}{P_{s}}\right)/\sqrt{T_{s}},chamber\begin{array}{c} = \end{array}b, \end{array}$$

where $P_{s}=6\times 10^{5}$ ${N/m}^{2}$ is the supply pressure; $P_{e}=1\times 10^{5}$ ${N/m}^{2}$ is the exhaust pressure; $T_{a}$ and $T_{b}$ are the cylinder air temperature of chamber A and B, respectively. In Equation (20), $-K_{f}x_{2}-K_{s-c}(x_{1})S(x_{2},x_{3},x_{4}),$ denotes the sum of the effects of the system static and dynamic friction forces, and

(24) $$K_{s-c}(x_{1})S(x_{2},x_{3},x_{4}):=\left\{ \begin{array}{l} A(x_{3}-x_{4})\\ \;\mathrm{as}\;x_{2}=0\;\mathrm{and}\;\left|A(x_{3}-x_{4})\right|\leq K_{s}(x_{1})\\ K_{c}(x_{1})sign(x_{2})\\ \;\mathrm{as}\;x_{2}\neq 0\;\mathrm{or}\;\left|A(x_{3}-x_{4})\right|>K_{s}(x_{1}) \end{array}\right.$$

where A denotes the piston area of the cylinder; $K_{s}(x_{1})$ indicates the position-dependent static friction forces, and $K_{c}(x_{1})$ is the variable position-dependent load due to friction. 

### 4.2. Input–Output Feedback Linearization

According to [36], an arbitrary nonlinear single-input single-output system can be linearized by differentiating its output. By applying feedback linearization theory to an RPA and neglecting the static frictional forces in Equation (20), the system can be expressed as:

(25) $$\begin{array}{l} \dot{x}=f(x)+g(x)u\\ y=h(x)=x_{1} \end{array}$$

where the state vector $x\equiv {\left[x_{1}\;x_{2}\;x_{3}\;x_{4}\right]}^{T}$ and u are, respectively, defined as the spool displacement of PDCV and the control input, and the corresponding vector fields f(x) and g(x) are described as:

(26) $$f(x)=\left[\begin{array}{c} x_{2}\\ \frac{1}{M}\left[-K_{f}x_{2}+A(x_{3}-x_{4})\right]\\ \frac{-k(x_{2}x_{3})}{x_{1}+\Delta }\\ \frac{k(x_{2}x_{4})}{l-x_{1}+\Delta } \end{array}\right]$$

and

(27) $$g(x)=\left[\begin{array}{c} 0\\ 0\\ \frac{kRT_{S}C_{d}C_{0}wf(x_{3},P_{s},P_{e})}{A(x_{1}+\Delta )}\\ \frac{-kRT_{S}C_{d}C_{0}wf(x_{4},P_{s},P_{e})}{A(l-x_{1}+\Delta )} \end{array}\right]$$

where f(x) and g(x) are partially unknown and smooth vector functions. After linearization, Equation (20) becomes:

(28) $$y^{\left(3\right)}(t)=F(x)+G(x)u+d(x)$$

where

(29) $$F(x)=\frac{K_{f}^{2}x_{2}-K_{f}A(x_{3}-x_{4})}{M^{2}}-\frac{Akx_{2}[x_{3}(l-x_{1}+\Delta )+x_{4}(x_{1}+\Delta )]}{M(x_{1}+\Delta )(l-x_{1}+\Delta )}$$

(30) $$G(x)=\frac{kRT_{s}C_{d}C_{0}w[\hat{f}(x_{3},P_{s},P_{e})(l-x_{1}+\Delta )+\hat{f}(x_{4},P_{s},P_{e})(x_{1}+\Delta )]}{M(x_{1}+\Delta )(l-x_{1}+\Delta )}$$

$x(t)={\left[\begin{array}{ccc} y(t) & \dot{y}(t) & \ddot{y}(t) \end{array}\right]}^{T}\in R^{3}$ is the state vector; u∈R and y∈R are the control input and the system output, respectively; d(x) denotes the external disturbance and the unmodeled friction force of the piston. It is assumed that |d(x)|≤D for all states x(t), and F(x) and G(x) are partially unknown functions with uncertain time-varying parameters. Without loss of generality, G(x) can be assumed to be strictly positive. Evidently, an additional disturbance compensator is necessary to take account of the lump of disturbances. 

### 4.3. Development of Control Strategy AIT2FC-STFSMC

**Figure 5:**
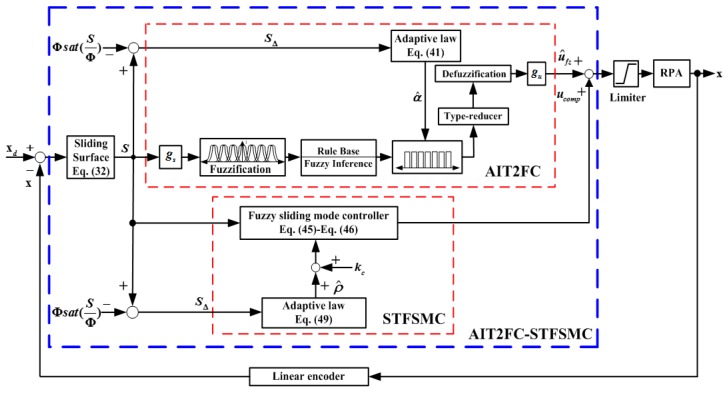
Overall system for the RPA-driven 3-DOF TPM.

As to the control strategy, the proposed AIT2FC-STFSMC is to attenuate disturbances and track trajectories for the RPA-driven 3-DOF TPM with high nonlinearity and time variation. Figure 5 shows the relationship between the AIT2FC and the STFSMC. In the AIT2FC-STFSMC, the adaptive interval type-2 fuzzy controller (AIT2FC) is designed as a trajectories tracking controller, where the interval type-2 fuzzy system is used to mimic an ideal controller. However, an approximation error may happen when using the AIT2FC. Hence, the self-tuning fuzzy sliding-mode compensator (STFSMC) is derived to compensate for the difference between the ideal controller and AIT2FC, as well as external disturbances. The AIT2FC is able to automatically adjust the fuzzy rules and reduce the number of fuzzy rules. Nevertheless, the boundary of approximation error is very difficult to measure in industrial applications. A large pre-set boundary will lead to a large chattering phenomenon in control output and will wear the bearing mechanism and excite unstable dynamics. Otherwise, a small boundary may make the controlled system unstable. To overcome the above-mentioned problem, a simple estimation algorithm is investigated to observe the boundary of approximation error in real-time. According to the on-line adjustment for the boundary, the chattering in control output can be much reduced.

The reference signals are defined as $x_{d}(t)={\left[\begin{array}{cccc} y_{d}(t) & {\dot{y}}_{d}(t) & \cdots & y_{d}^{\left(n-1\right)}(t) \end{array}\right]}^{T},$ so the tracking error vector is expressed as:

(31) $$e(t)=x_{d}(t)-x(t)={\left[e(t)\;\dot{e}(t)\;\ldots e^{\left(n-1\right)}(t)\right]}^{T}$$

The sliding surface is:

(32) $$S(t)=c_{1}e(t)+c_{2}\dot{e}(t)+\ldots +e^{\left(n-1\right)}(t)$$

where $c_{i}$ is specified such that $\sum_{i=1}^{n}c_{i}\lambda^{i-1}$ is a Hurwitz polynomial and λ is a Laplace operator. If the functions F(x) and G(x) in Equation (28) are known and the external disturbance d(x) is measurable, the ideal control law is derived as:

(33) $$u^{*}=\frac{1}{G(x)}[\eta S_{\Delta }(t)+\Lambda_{s}^{T}e(t)-F(x)-d(x)+y_{d}^{\left(n\right)}]$$

where η>0 is a constant; $\Lambda_{s}={\left[0,c_{1},c_{2},\cdots ,c_{n-2}\right]}^{T}$ is the constant vector, and $S_{\Delta }(t)=S-\Phi sat(S/\Phi )$, for which Φ≥0 is the width of the boundary layer of the sliding surface S. The properties of the function $S_{\Delta }$ are described as follows:

Property 1: As |S(t)|>Φ then $\left|S_{\Delta }(t)\right|=\left|S(t)\right|-\Phi$ and ${\dot{S}}_{\Delta }(t)=\dot{S}(t)$.
Property 2: As |S(t)|≤Φ then $S_{\Delta }(t)={\dot{S}}_{\Delta }(t)=0$.

These properties of the boundary layer are applied in the design of the controller, such that the adaptation terminates as soon as the boundary layer is reached to avoid the possibility of unbounded growth. Differentiating Equation (32) gives:

(34) $$\dot{S}(t)=\Lambda_{s}^{T}e(t)+x_{d}^{\left(n\right)}-F(x)-G(x)u-d(x)$$

Substituting Equation (33) into Equation (34) gives:

(35) $$\dot{S}(t)+\eta S_{\Delta }(t)=0$$

The convergence for $e(t)={\left[e(t)\;\dot{e}(t)\;\ldots e^{\left(n-1\right)}(t)\right]}^{T}$ in Equation (35) can be achieved for η>0. However, some variables in Equations (29) and (30) may be unknown or perturbed, and d(x) may not be measurable. Thus, it is difficult to obtain a precise model for the functions F(x) and G(x), and the implementation of the ideal control law $u^{*}$ is also impossible for the RPA. In this regard, the AIT2FC yields ${\hat{u}}_{fz}$ to approximate the ideal control law and combines the STFSMC with $u_{comp}(S)$ to compensate for the disturbance and modeling error. The proposed control law is:

(36) $$u={\hat{u}}_{fz}(S,\hat{\alpha })+u_{comp}(S)$$

### 4.4. Design of the AIT2FC

In this section, a single-input AIT2FC is used to formulate the control law ${\hat{u}}_{fz}$. For the AIT2FC, the *i*th fuzzy rule is:

(37) $$R^{i}:if\;S\;is\;{\tilde{F}}_{T_{2}S}^{i}\;then\;{\hat{u}}_{fz}\;is\;{\hat{\alpha }}_{T_{2}fz}^{i},\;(i=1,\ldots ,M)$$

where S is the input variable; ${\tilde{F}}_{T_{2}S}^{i}$ is an interval type-2 fuzzy set; ${\hat{\alpha }}_{T_{2}fz}^{i}$ is an interval type-2 singleton fuzzy set, and M is the number of rules. Using singleton fuzzification, product inference, and center-average defuzzification, the output of the AIT2FC is:

(38) $${\hat{u}}_{fz}(S,\hat{\alpha })=\frac{y_{l}+y_{r}}{2}=\frac{1}{2}\left[\begin{array}{cc} {\hat{\alpha }}_{l}^{T} & {\hat{\alpha }}_{r}^{T} \end{array}\right]\left[\begin{array}{l} \xi_{l}\\ \xi_{r} \end{array}\right]={\hat{\alpha }}^{T}\xi ,$$

where $y_{l}$ and $y_{r}$, respectively, represent the farthest left and the farthest right points of the interval type-2 set. In (38), the weight vector ${\hat{\alpha }}^{T}=\left[{\hat{\alpha }}_{1},{\hat{\alpha }}_{2},\cdots ,{\hat{\alpha }}_{M}\right]$ is used to estimate the optimal weight vector $\alpha^{*T}=\left[\alpha_{1}^{*},\alpha_{2}^{*},\cdots ,\alpha_{M}^{*}\right]$ and the parameter ${\hat{\alpha }}^{*T}$ is reasonably assumed to be bounded. The farthest left point of the interval type-2 set is defined as:

(39) $$\begin{array}{l} y_{l}=\frac{\sum_{i=1}^{L}{\bar{\mu }}_{F_{T_{2}S}^{i}}(S){\hat{\alpha }}_{l}^{i}+\sum_{i=L+1}^{M}{\underline{\mu }}_{F_{T_{2}S}^{i}}(S){\hat{\alpha }}_{l}^{i}}{\sum_{i=1}^{L}{\bar{\mu }}_{F_{T_{2}S}^{i}}(S)+\sum_{i=L+1}^{M}{\underline{\mu }}_{F_{T_{2}S}^{i}}(S)}=\sum_{i=1}^{L}{\bar{p}}_{l}^{i}{\hat{\alpha }}_{l}^{i}+\sum_{i=L+1}^{M}{\underline{p}}_{l}^{i}{\hat{\alpha }}_{l}^{i}\\ \;=[\begin{array}{cc} {\bar{\hat{\alpha }}}_{l}^{T} & {\underline{\hat{\alpha }}}_{l}^{T} \end{array}]\left[\begin{array}{c} {\bar{p}}_{l}\\ {\underline{p}}_{l} \end{array}\right]={\hat{\alpha }}_{l}^{T}\xi_{l} \end{array}$$

where ${\bar{\mu }}_{F_{T_{2}S}^{i}}$ and ${\underline{\mu }}_{F_{T_{2}S}^{i}}$, respectively, represent the upper and lower degrees of the membership function. ${\bar{p}}_{l}^{i}={\bar{\mu }}_{F_{T_{2}S}^{i}}(S)/W_{l},$ and ${\underline{p}}_{l}^{i}={\underline{\mu }}_{F_{T_{2}S}^{i}}(S)/W_{l},$ in which $W_{l}=\sum_{i=1}^{L}{\bar{\mu }}_{F_{T_{2}S}^{i}}(s)+\sum_{i=L+1}^{M}{\underline{\mu }}_{F_{T_{2}S}^{i}}(s).$ The farthest right point of the interval type-2 set is defined as:

(40) $$\begin{array}{l} y_{r}=\frac{\sum_{i=1}^{R}{\bar{\mu }}_{F_{T_{2}S}^{i}}(S){\hat{\alpha }}_{r}^{i}+\sum_{i=R+1}^{M}{\underline{\mu }}_{F_{T_{2}S}^{i}}(S){\hat{\alpha }}_{r}^{i}}{\sum_{i=1}^{R}{\bar{\mu }}_{F_{T_{2}S}^{i}}(S)+\sum_{i=R+1}^{M}{\underline{\mu }}_{F_{T_{2}S}^{i}}(S)}=\sum_{i=1}^{R}{\bar{p}}_{r}^{i}{\hat{\alpha }}_{r}^{i}+\sum_{i=R+1}^{M}{\underline{p}}_{r}^{i}{\hat{\alpha }}_{r}^{i}\\ \;=[\begin{array}{cc} {\bar{\hat{\alpha }}}_{r}^{T} & {\underline{\hat{\alpha }}}_{r}^{T} \end{array}]\left[\begin{array}{c} {\bar{p}}_{r}\\ {\underline{p}}_{r} \end{array}\right]={\hat{\alpha }}_{r}^{T}\xi_{r}, \end{array}$$

where ${\hat{\alpha }}_{r}^{i}$ is farthest right point of ${\hat{\alpha }}_{T_{2}f_{z}}^{i}$, ${\bar{p}}_{r}^{i}={\bar{\mu }}_{F_{T_{2}S}^{i}}(S)/W_{r}$, and ${\underline{p}}_{r}^{i}={\underline{\mu }}_{F_{T_{2}S}^{i}}(S)/W_{r}$, in which $W_{r}=\sum_{i=1}^{R}{\bar{\mu }}_{F_{T_{2}S}^{i}}(s)+\sum_{i=R+1}^{M}{\underline{\mu }}_{F_{T_{2}S}^{i}}(s)$. The parameters L and R in (39) and (40), respectively, are calculated by using type reduction [37]. The adaptive law for the AIT2FC is given as:

(41) $$\dot{\hat{\alpha }}=\eta_{1}\cdot S_{\Delta }\cdot \xi ,$$

where $\eta_{1}>0$ is the adaptive learning rate.

### 4.5. Design of the STFSMC

**Figure 6:**
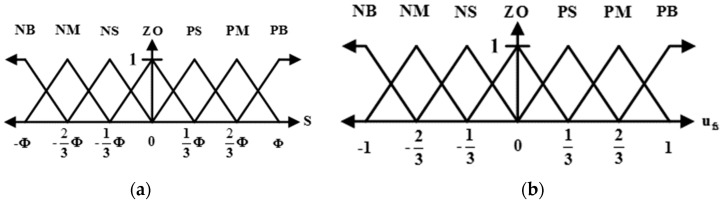
Membership functions for the fuzzy input S and the fuzzy output. (**a**) membership functions for the fuzzy input S; (**b**) membership functions for The fuzzy output $u_{fs}$.

The design objective of STFSMC is to compensate for the disturbances [38]. The sliding surface S is again specified as the input and the sliding control law $u_{fs}$ is the output. Figure 6 shows the membership functions and the associated linguistic variables as follows:

(42) $$\begin{array}{l} T(S)=\left\{NB,NM,NS,ZO,PS,PM,PB\right\}=\left\{f_{s}^{1},f_{s}^{2},f_{s}^{3},f_{s}^{4},f_{s}^{5},f_{s}^{6},f_{s}^{7}\right\},\\ T(u_{fs})=\left\{NB,NM,NS,ZO,PS,PM,PB\right\}=\left\{f_{u}^{1},f_{u}^{2},f_{u}^{3},f_{u}^{4},f_{u}^{5},f_{u}^{6},f_{u}^{7}\right\}, \end{array}$$

The fuzzy rules are simply expressed as:

(43) $$R^{l}:\;if\;S\;\mathrm{is}\;f_{s}^{l}\;then\;u_{fs}\;\mathrm{is}\;f_{u}^{8-l},\;l=1,\ldots ,7$$

By using singleton fuzzification, max-min inference, and center-average defuzzification, the output of STFSMC is:

(44) $$u_{fs}=\frac{\int_{-1}^{1}u_{fs}F(u_{fs})du_{fs}}{\int_{-1}^{1}F(u_{fs})du_{fs}}$$

where $F(u_{fs})=\overset{7}{\underset{l=1}{\vee }}\left[f_{s}^{l}(S)\wedge f_{u}^{8-l}(u_{fs})\right].$ To avoid substantial computational cost for the general fuzzy control algorithm, $u_{fs}$ can be calculated for two cases:

Case 1: k=S/Φ<0:

(45) $$u_{fs}=\left\{ \begin{array}{ccc} 1 & ,if & k<-1\\ \frac{7.5k^{2}+13.5k+5}{9k^{2}+15k+5} & ,if & -1\leq k<-\frac{2}{3}\\ \frac{9k^{2}+11k+2}{18k^{2}+18k+2} & ,if & -\frac{2}{3}\leq k<-\frac{1}{3}\\ \frac{1.5k^{2}+1.5k}{9k^{2}+3k-1} & ,if & -\frac{1}{3}\leq k<0 \end{array}\right.$$

Case 2: k=S/Φ≥0:

(46) $$u_{fs}=\left\{ \begin{array}{ccc} \frac{-1.5k^{2}+1.5k}{9k^{2}-3k-1} & ,if & 0\leq k<\frac{1}{3}\\ \frac{-9k^{2}+11k-2}{18k^{2}-18k+2} & ,if & \frac{1}{3}\leq k<\frac{2}{3}\\ \frac{-7.5k^{2}+13.5k-5}{9k^{2}-15k+5} & ,if & \frac{2}{3}\leq k<1\\ -1 & ,if & k\geq 1 \end{array}\right.$$

where Φ>0 is given to denote the width of the boundary layer. It can be verified that $u_{fs}=-\mathrm{sgn}(S)$ as |S|≥Φ, and, thus, the STFSMC is designed as:

(47) $$u_{comp}(S)=-(k_{c}+\hat{\rho })u_{fs}$$

where $k_{c}$ is a compensation gain, which is:

(48) $$k_{c}=\frac{M_{2}(x)\left|S_{\Delta }(t)\right|}{2M_{0}^{2}(x)}$$

where $M_{0}(x)$ and $M_{2}(x)$ are specified variables. $\hat{\rho }$ is an adaptive compensation gain [38] that is represented as:

(49) $$\dot{\hat{\rho }}=\eta_{2}\cdot \left|S_{\Delta }\right|$$

where $\eta_{2}>0$ is a learning rate that is greater than zero. Figure 5 illustrates the overall system for the proposed AIT2FC-STFSMC. For the AIT2FC, the parameter $g_{s}$ is used to ensure that the sliding surface S is within the range of the fuzzy input and the gain factor $g_{u}$ is used to regulate the fuzzy output ${\hat{u}}_{fz}.$

**Assumption** **1.** *The control signal*u*is linear with respect to*$y^{\left(n\right)}$*and*G(x)≠0*for*x*in the controllable region*$U_{c}$.

**Assumption** **2.** *The internal dynamics of each RPA system are stable under the influence of the AIT2FC-STFSMC.*

**Assumption** **3.** *The boundary conditions,* $0<M_{0}(x)\leq$ $G(x)\leq M_{1}(x)$ *and* $\left|\dot{G}(x)\right|\leq M_{2}(x)$ *, hold, where* $M_{0}(x)$ *,* $M_{1}(x)$ *, and* $M_{2}(x)$ *are known functions.*

It is noted that Assumption 1 is needed for the design of the AIT2FC, Assumption 2 is needed for the both of the AIT2FC and the STFSMC, and Assumption 3 is needed for the design of the STFSMC.

**Theorem** **1.** *If the RPA is represented as the form of an affine system (28) that satisfies Assumptions 1, 2, and 3. By using the control law (36), where* ${\hat{u}}_{fz}(S,\hat{\alpha })$ *represents the AIT2FC (see Equation (38)) and* $u_{comp}(S)$ *represents the STFSMC (See Equation (47)), and the adaptive laws (41) and (49), it is observed that (i) the system state* x *and the control law* u *are bounded and (ii) the tracking errors converge to 0 as* t→∞.

**Proof.**  See Appendix A. □

## 5. Robot Vision for 3-DOF TPM

### 5.1. Image Capturing

In this study, images of an object were captured by a webcam and then transferred to LabVIEW through an API with image processing toolkits. The API *IMAQdx Open Camera* is used to open a video source in the RGB color model, in which the *Property Node* is a function applied to set the image resolution and the frames per second. *IMAQ Create* is an API applied to create a buffer for temporarily storing images. The images in the buffer are grabbed by the API *IMAQdx Grab* as requested. 

### 5.2. Define a Template Pattern

To define a template pattern, the captured image for an object in RGB is first converted to grayscale by color transformation, which is represented as:

(50) Y=0.299R+0.587G+0.114B,

A mask for the object is obtained by manually drawing the minimum rectangular region that contains the object. The region is termed the region of interest (ROI). The API *IMAQ ConstructROI VI* extracts image features within the ROI and creates the template pattern. The template pattern and its features for the targeted object are stored in a database. 

### 5.3. Pattern Recognition

**Figure 7:**
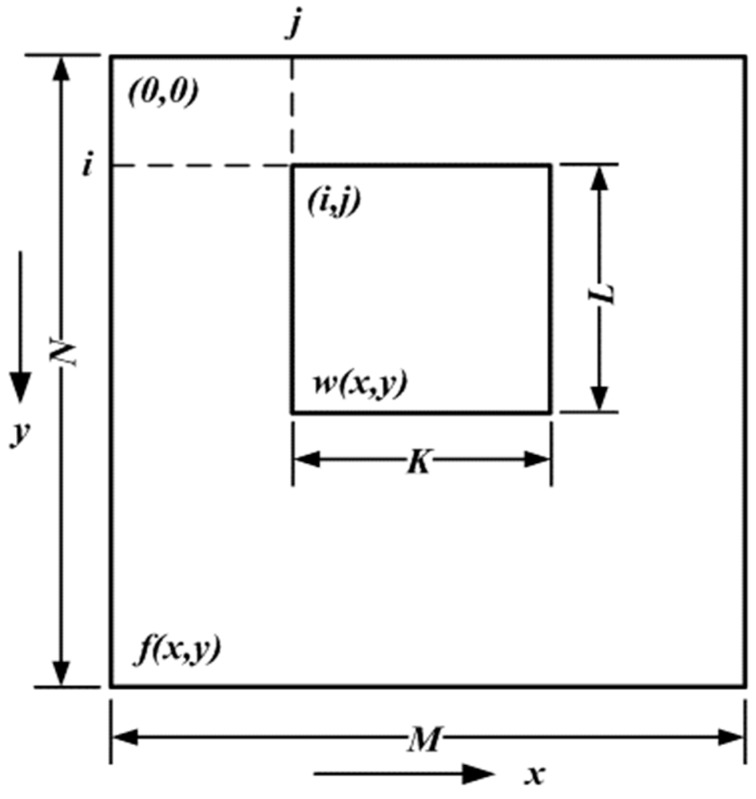
Block matching process.

In this paper, pattern recognition is applied to determine the location of an object that has the same image features as the template pattern. Once activated, the pattern recognition system captures an image and uses the *NI IMAQ Vision* API to search for patterns similar to the template over the input image by using block matching. Figure 7 indicates the processes of block matching, in which f(x,y) is the greyscale of the image f with the dimension M×N and w(x,y) is the greyscale of the image w(x,y) with the dimension K×L. It is noted that M≥K and N≥L. Block matching searches similar patterns from the pixel coordinate (1, 1) to (M−K,N−L) with a K×L dimension block, w(x,y), and calculates the relationship C(i,j) between f(i,j) and w(i,j) by Equation (51), which is:

(51) $$C(i,j)=\sum_{x=0}^{L-1}\sum_{y=1}^{K-1}w(x,y)f(x+i,y+j)$$

After searching is conducted, the pattern with the biggest value C is considered a candidate pattern. After that, its correlation is defined as:

(52) $$R(i,j)=\frac{\sum_{x=0}^{L-1}\sum_{y=0}^{K-1}\left[w(x,y)-\bar{w}\right][f(x+i,y+j)-f(i,j)]}{\sqrt{\sum_{x=0}^{L-1}\sum_{y=0}^{K-1}{\left[w(x,y)-\bar{w}\right]}^{2}}\sum_{x=0}^{L-1}\sum_{y=0}^{K-1}{\left[f(x+i,y+j)-f(i,j)\right]}^{2}}$$

The pattern is defined as the best matching pattern, especially if −1<R(i,j)<1. Image recognition is implemented by LabVIEW with three APIs as follows: (1) the *IMAQ Read Image and Vision Info VI* API calls the built template pattern; (2) The *IMAQ Match Pattern* API searches for the matches with respect to the preset matching parameters; (3) The *Unbundle by Name Function* API calculates the centroid of the optimal matching pattern, which represents the entirety of the optimal matching pattern with respect to the image.

### 5.4. Spatial Calibration 

Spatial calibration converts a pixel coordinate to a real-world coordinate. In the meantime, two APIs can be applied to compensate for the potential perspective errors and nonlinear distortions in the image, as introduced as follows:

(1) The *IMAQ Calibration Target to Points—Circular Dots* API detects circular dots in a binary image and returns pixels and real-world points for calibration;
(2) The *IMAQ Get Calibration Info* API returns calibration information associated with the image.

After spatial calibration is performed, one can identify the coordinate of the target object in the real world.

## 6. Experiments and Discussion

The RPA is regulated by the PDCV. Instead of using acceleration sensors, in this study the velocity and acceleration are estimated by numerically differentiating the position and the velocity with respect to time. To reduce the signal disturbances during the numerical difference operation, a digital filter is used as follows:

(53) $$y_{out}\left(i\right)=0.7y_{out}\left(i-1\right)+0.15\left[y_{in}(i)+y_{in}(i-1)\right]$$

where $y_{out}(t)$ represents the filtered signal and $y_{in}(t)$ is the input data from the position measurement of the piston. The input voltage of the PDCV is applied as the control signal. The membership functions for S and $u_{fs}$ are displayed in Figure 5.

### 6.1. Experiment 1(Trajectory Tracking—Square Trajectory)

**Figure 8:**
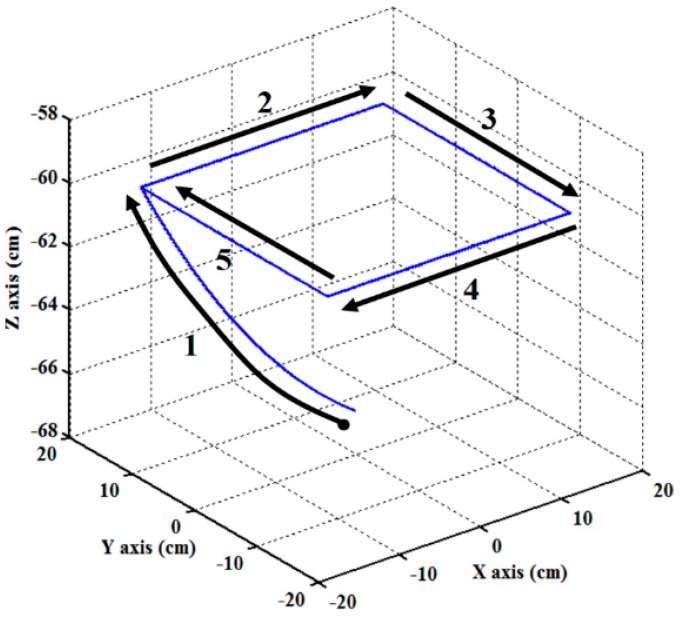
Designed square loop trajectory for the end-effector path tracking control experiment.

The square trajectory is often used in many practical applications, such as the pick-and-place operation. It can be segmented by five segments and has four positioning points at the vertex of the square. The profile and the moving direction of the square trajectory are illustrated in Figure 8. Segment 1 is modeled as a fifth-order trajectory [27,33], and segments 2 to 5 are modeled as a line. In the experiment, at the beginning, the end-effector moves from the initial position (0,0,−66.6 cm) to (−15 cm,15 cm,−60 cm) in 3 s. Then, the end-effector moves along the square loop path with the edge length of 30 cm and back to the positioning point (−15 cm,15 cm,−60 cm) in 6 s. To effectively control the RPAs, the trajectories for each RPA are also modeled as a fifth-order polynomial function [27,33] with zero initial and final velocities as well as zero initial and final accelerations.

**Figure 9:**
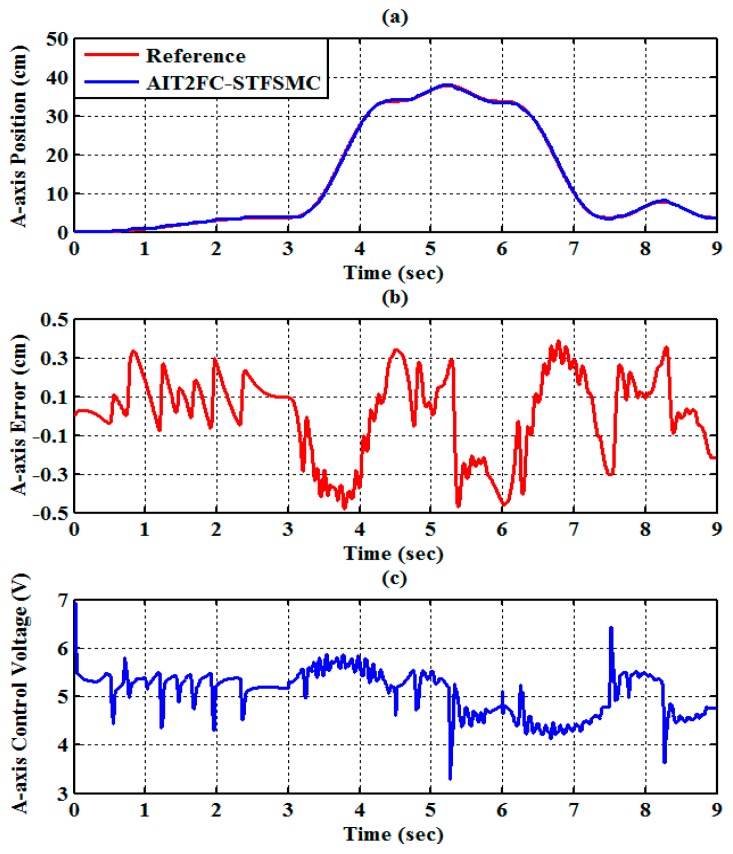
Experimental results of end-effector path tracking for an A-axis RPA via a square loop trajectory (**a**) tracking response, (**b**) tracking error, and (**c**) control voltage.

**Figure 10:**
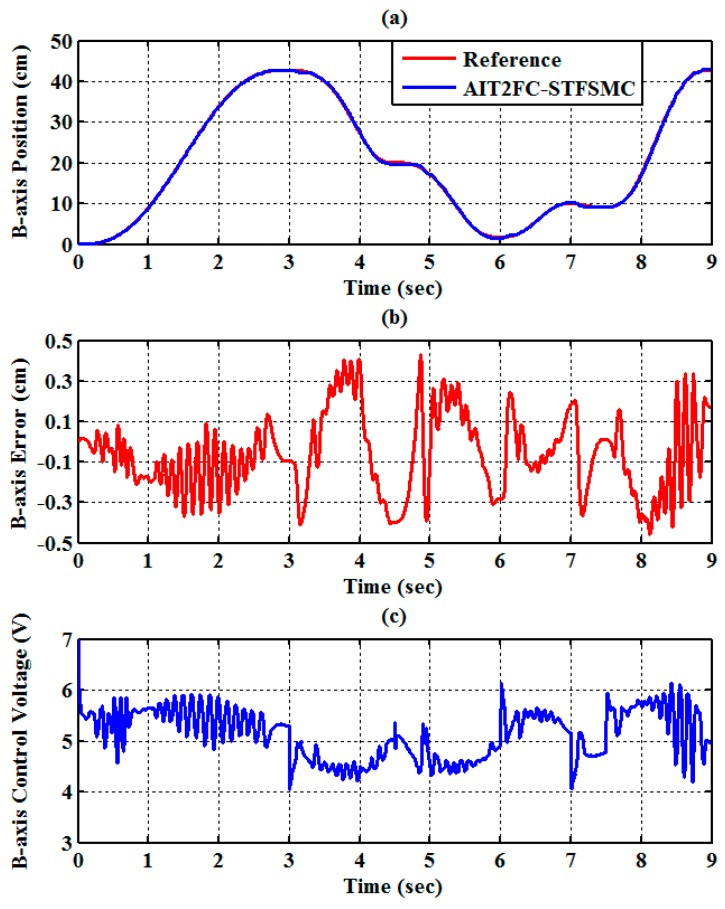
Experimental results of end-effector path tracking for a B-axis RPA via a square loop trajectory (**a**) tracking response, (**b**) tracking error, and (**c**) control voltage.

**Figure 11:**
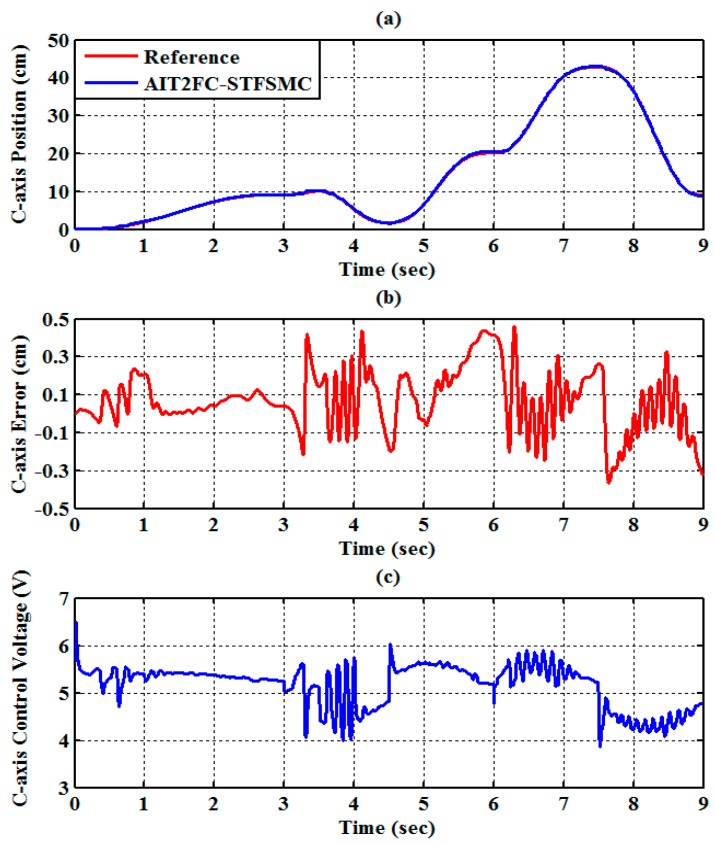
Experimental results of end-effector path tracking for a C-axis RPA via a square loop trajectory (**a**) tracking response, (**b**) tracking error, and (**c**) control voltage.

**Figure 12:**
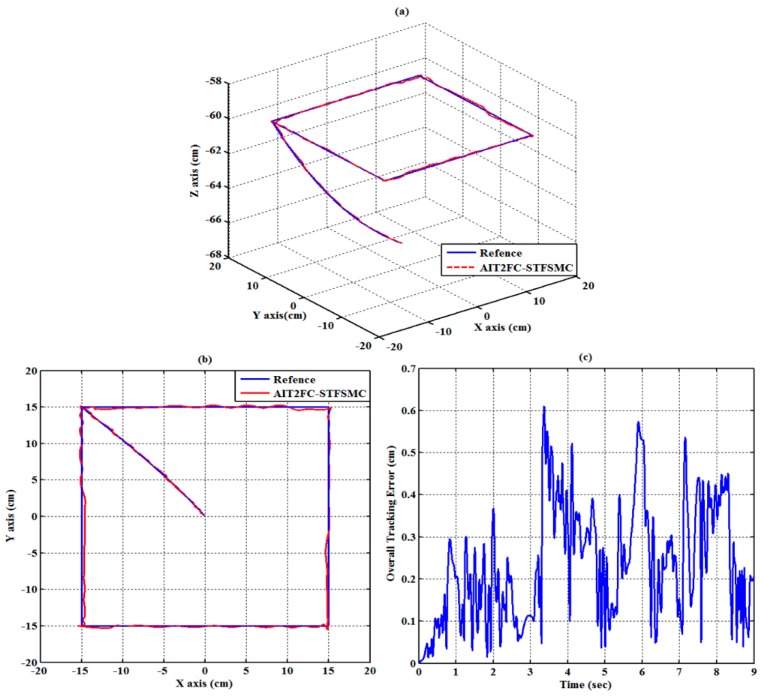
Estimated end-effector position tracking response for the square loop trajectory (**a**) calculated end-effector position (front view), (**b**) calculated end-effector position (top view), and (**c**) calculated end-effector position error.

The experimental results of the RPA response for each axis are shown in Figure 9, Figure 10 and Figure 11. The estimated end-effector position calculated from the forward kinematics is shown in Figure 12a,b. The estimated position error of the end-effector is calculated from the position error of the actuated joint, as shown in Figure 12c. The root-mean-square error (RMSE) of the path tracking control for the cylinder A, B, and C axes are 0.214, 0.203, and 0.173 cm, respectively. The maximum estimated error of the end-effector position is approximately 0.6097 at 3.37 s. 

### 6.2. Experiment 2 (Trajectory Tracking-Star Trajectory)

**Figure 13:**
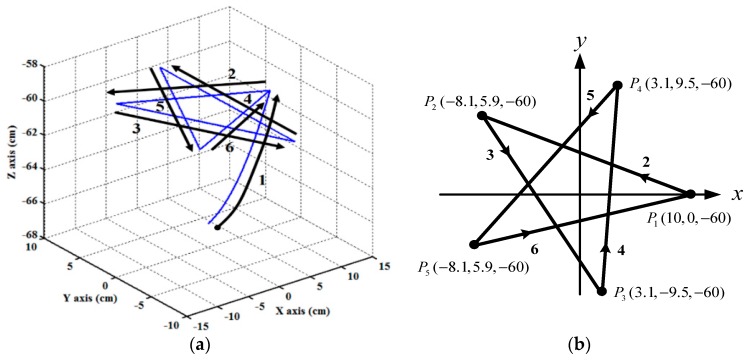
Designed star loop trajectory for the end-effector path tracking control experiment. (**a**) 3D star-motion trajectory; (**b**) top-view of the 3D star-motion trajectory.

**Figure 14:**
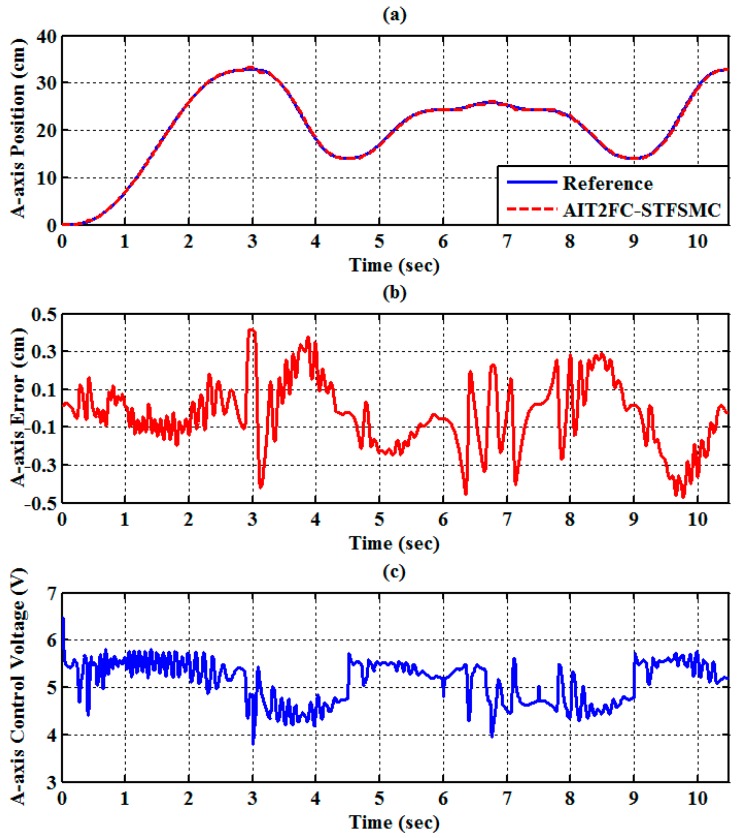
Experimental results of end-effector path tracking for the A-axis RPA via a star loop trajectory (**a**) tracking response, (**b**) tracking error, and (**c**) control voltage.

**Figure 15:**
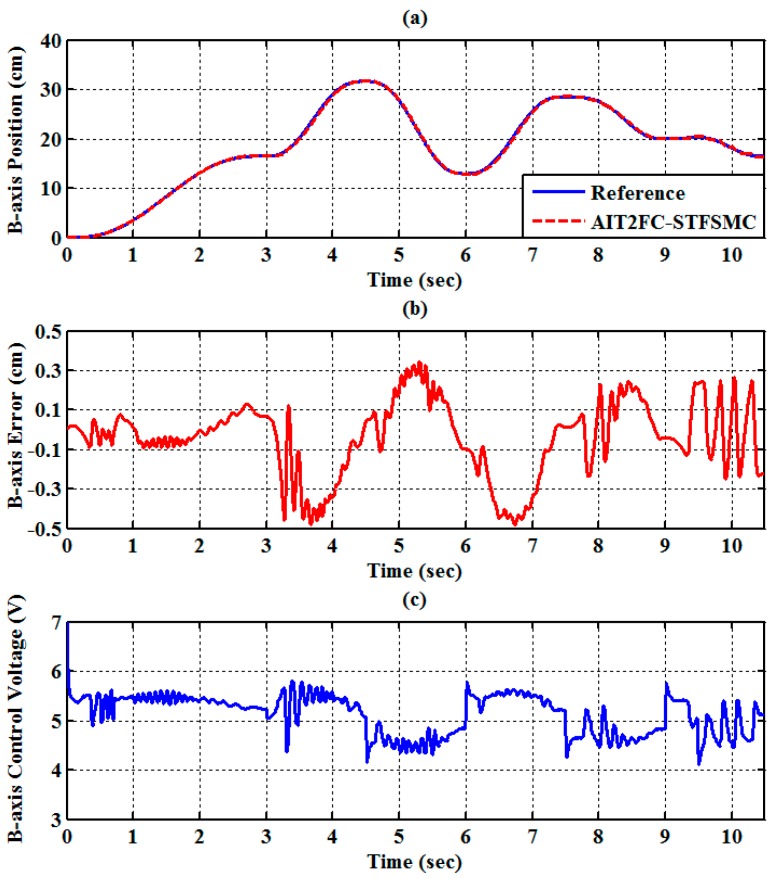
Experimental results of end-effector path tracking for the B-axis RPA via a star loop trajectory (**a**) tracking response, (**b**) tracking error, and (**c**) control voltage.

**Figure 16:**
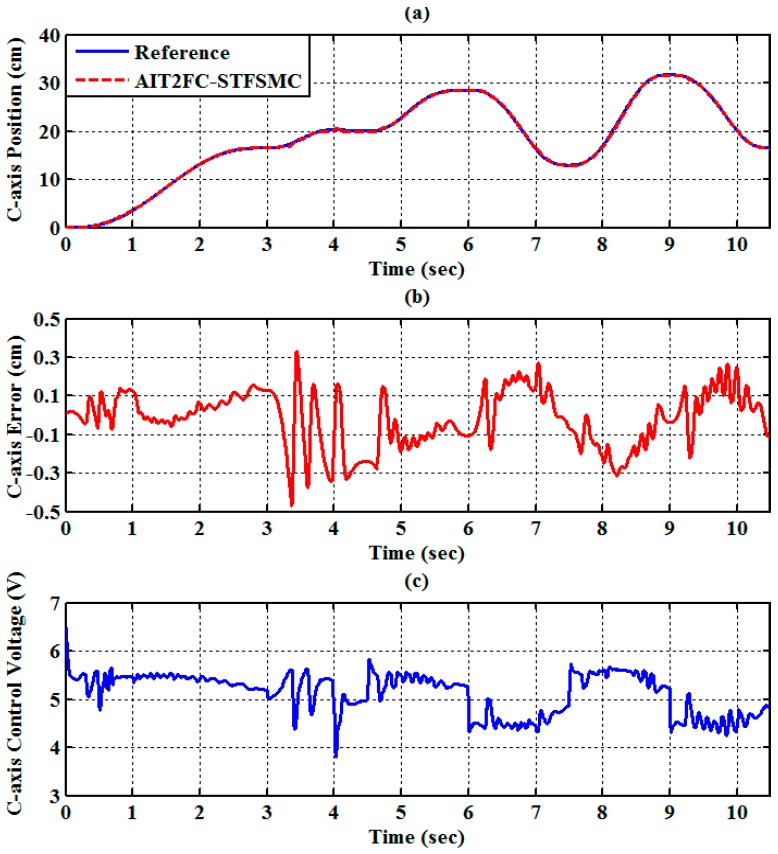
Experimental results of end-effector path tracking for the C-axis RPA via a star loop trajectory (**a**) tracking response, (**b**) tracking error, and (**c**) control voltage.

**Figure 17:**
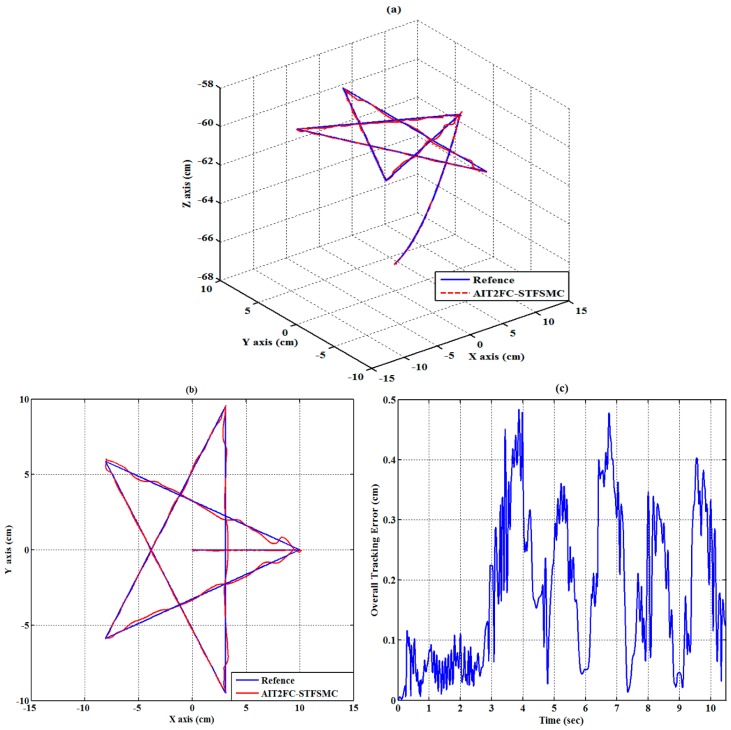
Estimated end-effector position tracking response for the star loop trajectory (**a**) calculated end-effector position (front view), (**b**) calculated end-effector position (top view), and (**c**) calculated end-effector position error.

In Experiment 2, a star motion is set as a reference 3D motion trajectory. The star trajectory is composed of six segments. The profile and moving direction of the designed trajectory are illustrated in Figure 13. Segment 1 is modeled as a fifth-order trajectory [27,33] from the initial position (0,0,−66.6 cm) to (10 cm,0 cm,−60 cm), and segments 2 to 6 are modeled as a straight line. At the beginning, the end-effector moves along segment 1 in 3 s. Then, the end-effector moves sequentially along segment 2 to segment 6, where segment 2 is a straight line from $P_{1}$ (10 cm,0 cm,−60 cm) to $P_{2}$ (−8.1 cm,5.9 cm,−60 cm), segment 3 is a straight line from $P_{2}$ (−8.1 cm,5.9 cm,−60 cm) to $P_{3}$ (3.1 cm,−9.5 cm,−60 cm), segment 4 is a straight line from $P_{3}$ (3.1 cm,−9.5 cm,−60 cm) to $P_{4}$ (3.1 cm,9.5 cm,−60 cm), segment 5 is a straight line from $P_{4}$ (3.1 cm,9.5 cm,−60 cm) to $P_{5}$ (−8.1 cm,−5.9 cm,−60 cm), and segment 6 is a straight line from $P_{5}$ (−8.1 cm,−5.9 cm,−60 cm) to $P_{1}$ (10 cm,0 cm,−60 cm). The traveling duration for each of segments 2 to 6 is set to 2 s. To effectively control the RPAs, the trajectories for each RPA are also modeled as a fifth-order polynomial function [27,33] with zero initial and final velocities as well as zero initial and final accelerations. The experimental results of the RPA response for each axis are presented in Figure 14, Figure 15 and Figure 16. The estimated end-effector position calculated from the forward kinematics is depicted in Figure 17a,b The estimated position error of the end-effector is calculated from the position error of the actuated joint and illustrated in Figure 17c. The RMSE values on the path tracking control of cylinders of A, B, and C axes are 0.176 cm, 0.189 cm, and 0.142 cm, respectively. The maximum estimated error of the end-effector position is approximately 0.4833 cm at 3.87 s.

### 6.3. Experiment 3 (Robot Vision)

#### 6.3.1. Stationary Object Localization

**Figure 18:**
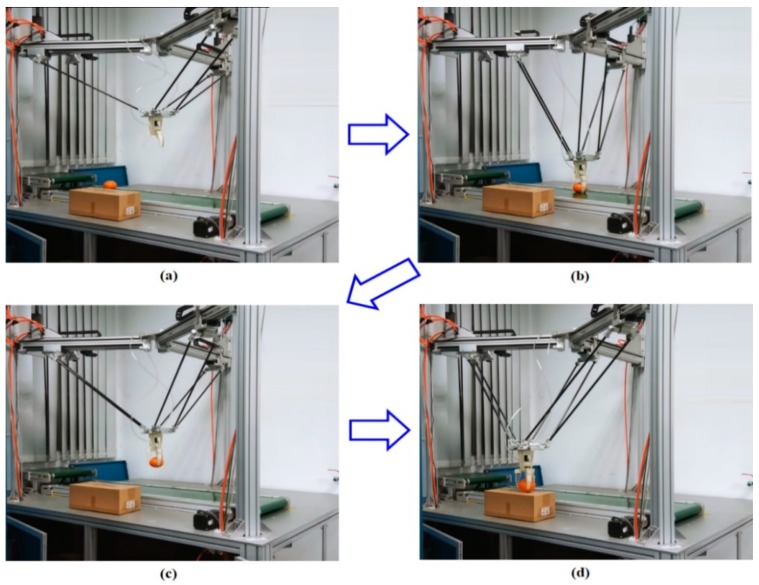
Pick-and-place experiment. (**a**) an object is on the conveyor belt; (**b**) the robot vision system locates the object and the PM picks it up; (**c**) the PM moves the object to the desired location (**d**) the PM puts the object down to the desired location.

This experiment measured the accuracy of the object-located technique for a stationary object. In the first experiment, a hexagonal object was placed on the conveyor belt, and the power was turned off. By using the presented robot vision after repeating this experiment 10 times, the average, variation, and maximum of the error for the *x*-coordinate, *y*-coordinate, and angle were discovered and are shown in Table 1. The variations of the *x*-coordinate and *y*-coordinate were less than 0.03 cm, and the variation of the angle was less than 0.25°, which are within the position tolerance error for the robot arm. Table 1 shows the measuring accuracy of the visual camera. The maximum error of the *x*-coordinate and *y*-coordinate are, respectively, around 0.05 cm and 0.08 cm, as well as the maximum error of the angle which was around 0.09°. In this paper, the spatial calibration is applied to convert a pixel coordinate to a real-world coordinate. Clearly, the measuring error in the pixel coordinate will produce an error in the real-world coordinate after coordinate transformation. Furthermore, the error in the real-world coordinate will transfer to the motion control for the RPAs. In this paper, a soft pneumatic gripper installed on the end-effector is soft and triangular with hard crossbeams, so that it can buckle and deform in to conform around an object. It is able to easily grip an object with the radius between the interval of [35 mm 65 mm]. That is, if the motion-error, which may come from the measuring error, happens in trajectory tracking control for the RPAs, the soft pneumatic gripper can still successfully pick the object up. In our design, Experiment 3 shows the vision-based RPA-driven TPM accurately locates the object and successfully executes the pick-and-place operation, as shown in Figure 18.

#### 6.3.2. Pick-and-Place Operation

In this experiment, the conveyor belt moved at a fixed speed of 2.1 cm/s along the y-axis of the image. The conveyor belt conveyed a targeted object at a speed of 2.1 cm/s. The proposed robot-vision system estimated the location of the moving object by calculating the velocity formula. In the physical test, the estimated error of the location of the moving object was approximately 0.1 cm when the conveyor belt moved the object 20 cm along the y-axis of the image. Figure 18 illustrates a pick-and-place experiment. Figure 18a illustrates an object on the conveyor belt. After the power was connected to the conveyor belt, the conveyor belt conveyed the object along the *y*-axis of the image. The image of the moving object was captured by the webcam. Figure 18b depicts the robot vision system locating the object and the PM picking it up. The PM moved the object to the desired location, as illustrated in Figure 18c,d.

## 7. Conclusions

This study developed and implemented a vision-based RPA-driven 3-DOF TPM, which not only allows 3D path tracking control in a full-scale test rig but also provides robot vision to locate an object for vision-based operations. The AIT2FC-STFSMC was developed to allow path tracking control for the RPAs, in which the AIT2FC approximates the ideal control law and the STFSMC attenuates disturbances and uncertainties. The system’s webcam collected images of objects and transferred them to LabVIEW for image processing. Two types of experiments were conducted to confirm the feasibility of the proposed system. First, the evidence demonstrated that the end-effector of the manipulator successfully tracks the two given complex 3D trajectories under the RMSE of 0.22 cm. Second, two experiments were conducted on the vision-based RPA-driven 3-DOF TPM. The first experiment proved that the robot-vision system accurately located a stationary object, and the second experiment confirmed that the TPM successfully completed the pick-and-place operation for a moving object. 

## 8. Patents

There are two Taiwan utility model patents resulting from the developed PM, which are (1) Pneumatic-Actuator 3-DOF Translational Parallel Manipulator with the patent number of M463182; (2) Pneumatic-Actuator 3-DOF Translational Parallel Manipulator with Computer Vision with the patent number of M470742. 

## Figures and Tables

**Table 1:** Results of the robot vision test for the stationary object.

	Error Terms	*x*-Coordinate (cm)	*y*-Coordinate (cm)	Angle (degree)
Items				
Max. of Error		0.051186	0.078865	0.085908
Var. of Error		0.014741	0.022723	0.024787

## Appendix A

This Theorem can be proven using two steps. In Step 1, the system state x and the adaptive control u are bounded when the AIT2FC-STFSMC is used for the RPA. Next, one can calculate that the tracking error will converge to zero asymptotically in Step 2.

Step 1: Substituting Equations (33) and (36) into (34) yields

(A1) $$\dot{S}(t)+\eta S_{\Delta }(t)=G\left[u^{*}-{\hat{u}}_{fz}(S,\hat{\alpha })-u_{comp}(S)\right].$$

If Assumption 1 holds for Equation (A1), a candidate Lyapunov function is specified as:

(A2) $$V(S_{\Delta }(t),\tilde{\alpha },\tilde{\rho })=\frac{1}{2}\frac{S_{\Delta }^{2}}{G(x)}+\frac{1}{2\eta_{1}}{\tilde{\alpha }}^{T}\tilde{\alpha }+\frac{1}{2\eta_{2}}{\tilde{\rho }}^{2},$$

where ${\tilde{\alpha }}^{T}=\alpha^{*T}-{\hat{\alpha }}^{T}$ and $\tilde{\rho }=\rho^{*}-\hat{\rho }$ are, respectively, represented as the approximation errors of the parameter vectors ${{\tilde{\alpha }}^{*}}^{T}$ and $\rho^{*}$. $\eta_{1}$ and $\eta_{2}$ are positive constants. Differentiating Equation (A2) with respect to time yields:

(A3) $$\dot{V}(S_{\Delta },\tilde{\alpha },\tilde{\rho })=\frac{S_{\Delta }{\dot{S}}_{\Delta }}{G(x)}-\frac{\dot{G}(x)S_{\Delta }^{2}}{2G^{2}(x)}+\frac{1}{\eta_{1}}{\tilde{\alpha }}^{T}\dot{\tilde{\alpha }}+\frac{1}{\eta_{2}}\tilde{\rho }\dot{\tilde{\rho }},$$

If |S|≤Φ, then $S_{\Delta }(t)={\dot{S}}_{\Delta }(t)=0$. It follows $\dot{V}(S_{\Delta },\tilde{\alpha },\tilde{\rho })=0$. If |S|>Φ, then ${\dot{S}}_{\Delta }=\dot{S}$. Substituting Equation (A3) with Equation (A1) yields:

(A4) $$\begin{array}{l} \dot{V}(S_{\Delta },\tilde{\alpha },\tilde{\rho })=-\eta \frac{{S_{\Delta }}^{2}}{G(x)}-\frac{\dot{G}(x)S_{\Delta }^{2}}{2G^{2}(x)}+S_{\Delta }[u^{*}-{\hat{u}}_{fz}(S,\hat{\alpha })]-\frac{1}{2}S_{\Delta }u_{comp}(S)+\frac{1}{\eta_{1}}{\tilde{\alpha }}^{T}\dot{\tilde{\alpha }}+\frac{1}{\eta_{2}}\tilde{\rho }\dot{\tilde{\rho }}\\ =-\eta \frac{{S_{\Delta }}^{2}}{G(x)}-\frac{\dot{G}(x)S_{\Delta }^{2}}{2G^{2}(x)}+S_{\Delta }[u^{*}-{\hat{u}}_{fz}(S,\alpha^{*})+{\hat{u}}_{fz}(S,\alpha^{*})-{\hat{u}}_{fz}(S,\hat{\alpha })]+S_{\Delta }k_{c}u_{fs}+S_{\Delta }\hat{\rho }u_{fs}-\\ \frac{1}{\eta_{1}}{\tilde{\alpha }}^{T}\dot{\hat{\alpha }}-\frac{1}{\eta_{2}}\tilde{\rho }\dot{\hat{\rho }}\leq -\eta \frac{{S_{\Delta }}^{2}}{M_{1}(x)}+\frac{M_{2}(x)S_{\Delta }^{2}}{2M_{0}^{2}(x)}+\left|S_{\Delta }\right|\left|u^{*}-{\hat{u}}_{fz}(S,\alpha^{*})\right|+S_{\Delta }[{\hat{u}}_{fz}(S,\alpha^{*})-{\hat{u}}_{fz}(S,\hat{\alpha })]+\\ S_{\Delta }k_{c}u_{fs}+S_{\Delta }\hat{\rho }u_{fs}-\frac{1}{\eta_{1}}{\tilde{\alpha }}^{T}\dot{\hat{\alpha }}-\frac{1}{\eta_{2}}\tilde{\rho }\dot{\hat{\rho }}. \end{array}$$

Because $S_{\Delta }u_{fs}=-\left|S_{\Delta }\right|$, Equation (A4) is derived as:

(A5) $$\begin{array}{l} -\eta \frac{{S_{\Delta }}^{2}}{M_{1}(x)}+\left|S_{\Delta }\right|[\frac{M_{2}(x)\left|S_{\Delta }\right|}{2M_{0}^{2}(x)}-k_{c}]+\left|S_{\Delta }\right|\left|u^{*}-{\hat{u}}_{fz}(S,\alpha^{*})\right|+S_{\Delta }[{\hat{u}}_{fz}(S,\alpha^{*})-{\hat{u}}_{fz}(S,\hat{\alpha })]-\left|S_{\Delta }\right|\hat{\rho }-\frac{1}{\eta_{1}}{\tilde{\alpha }}^{T}\dot{\hat{\alpha }}-\frac{1}{\eta_{2}}\tilde{\rho }\dot{\hat{\rho }}\\ =-\eta \frac{{S_{\Delta }}^{2}}{M_{1}(x)}+\left|S_{\Delta }\right|[\frac{M_{2}(x)\left|S_{\Delta }\right|}{2M_{0}^{2}(x)}-k_{c}]+\left|S_{\Delta }\right|(\rho^{*}-\hat{\rho })+S_{\Delta }({\alpha^{*}}^{T}-{\hat{\alpha }}^{T})\xi -\frac{1}{\eta_{1}}{\tilde{\alpha }}^{T}\dot{\hat{\alpha }}-\frac{1}{\eta_{2}}\tilde{\rho }\dot{\hat{\rho }}. \end{array}$$

Then, because ${\dot{\tilde{\alpha }}}^{T}=-{\dot{\hat{\alpha }}}^{T}$ and $\dot{\tilde{\rho }}=-\dot{\hat{\rho }},$ Equation (A5) becomes:

(A6) $$\begin{array}{l} -\eta \frac{{S_{\Delta }}^{2}}{M_{1}(x)}+\left|S_{\Delta }\right|[\frac{M_{2}(x)\left|S_{\Delta }\right|}{2M_{0}^{2}(x)}-k_{c}]+\left|S_{\Delta }\right|\tilde{\rho }+S_{\Delta }{\tilde{\alpha }}^{T}\xi -\frac{1}{\eta_{1}}{\tilde{\alpha }}^{T}\dot{\hat{\alpha }}-\frac{1}{\eta_{2}}\tilde{\rho }\dot{\hat{\rho }}=-\eta \frac{{S_{\Delta }}^{2}}{M_{1}(x)}+\left|S_{\Delta }\right|[\frac{M_{2}(x)\left|S_{\Delta }\right|}{2M_{0}^{2}(x)}-k_{c}]+\\ {\tilde{\alpha }}^{T}(S_{\Delta }\xi -\frac{1}{\eta_{1}}\dot{\hat{\alpha }})+\tilde{\rho }(\left|S_{\Delta }\right|-\frac{1}{\eta_{2}}\dot{\hat{\rho }}). \end{array}$$

Substituting Equation (41) and (47)–(49) into Equation (A6) yields:

(A7) $$\dot{V}(S_{\Delta },\tilde{\alpha },\tilde{\rho })\leq -(\eta /M_{1}(x))\cdot {S_{\Delta }}^{2}.$$

From Equation (A7), $S_{\Delta }(t)$, $\tilde{\alpha }(t)$ and $\tilde{\rho }(t)$ are bounded if the initial values of $S_{\Delta }(0)$, $\tilde{\alpha }(0)$ and $\tilde{\rho }(0)$ are bounded. If $S_{\Delta }(t)$, $\tilde{\alpha }(t)$, and $\tilde{\rho }(t)$ are bounded, it implies that e(t) is bounded. Consequently, all of the system states x(t) are bounded, and the adaptive control u(t) is also bounded.

Step 2: From Equation (A7), the designed control law, including Equations (38), (41), (49), and (50) can guarantee that $S_{\Delta }\in L_{\infty }$ and the error converge. Integrating both sides of Equation (A7) yields:

(A8) $$\int_{0}^{\infty }S_{\Delta }^{2}dt\leq \frac{V(S_{\Delta }(\infty ),\tilde{\alpha },\tilde{\rho })-V(S_{\Delta }(0),\tilde{\alpha },\tilde{\rho })}{\eta /M_{1}(x)}$$

The right side of Equation (A8) is bounded, (i.e., $S_{\Delta }\in L_{\infty }$). Using Barbalat’s lemma [36], it is proven that $S_{\Delta }=0$ as t→∞, which means that the inequality |S|≤Φ holds and the tracking error e(t) will asymptotically converge to zero as t→∞.
